# Non-Criteria Obstetric Antiphospholipid Syndrome: How Different Is from Sidney Criteria? A Single-Center Study

**DOI:** 10.3390/biomedicines10112938

**Published:** 2022-11-15

**Authors:** Víctor M. Martínez-Taboada, Pedro Blanco-Olavarri, Sara Del Barrio-Longarela, Leyre Riancho-Zarrabeitia, Ana Merino, Alejandra Comins-Boo, Marcos López-Hoyos, José L. Hernández

**Affiliations:** 1 Division of Rheumatology, Hospital Universitario Marqués de Valdecilla-IDIVAL, 39008 Santander, Spain; 2 Departamento de Medicina y Psiquiatría, Universidad de Cantabria, 39011 Santander, Spain; 3 Division of Obstetrics and Gynecology, Hospital Marqués de Valdecilla, 39008 Santander, Spain; 4 Rheumatology Department, Hospital Sierrallana-IDIVAL, 39300 Torrelavega, Spain; 5 Immunology Department, Hospital Universitario Marqués de Valdecilla-IDIVAL, 39008 Santander, Spain; 6 Departamento de Biología Molecular, Universidad de Cantabria, 39011 Santander, Spain; 7 Department of Internal Medicine, Hospital Universitario Marqués de Valdecilla-IDIVAL, 39008 Santander, Spain

**Keywords:** pregnancy, obstetric morbidity, fetal loss, antiphospholipid syndrome, antiphospholipid antibodies, non-criteria

## Abstract

This study aims to compare the demographic characteristics, clinical features, serology, and fetal–maternal outcomes between women with obstetric antiphospholipid syndrome (APS) and those with non-criteria (NC)-APS and seronegative (SN)-APS. Two-hundred and sixty-three women with APS obstetric morbidity ever pregnant were included. Of those, 66 met the APS classification criteria, 140 were NC-APS, and 57 were SN-APS. Patients with other autoimmune diseases were excluded. Adverse pregnancy outcomes (APO) included early pregnancy loss, fetal death, preeclampsia, abruptio placentae, and preterm birth. The mean age of the study group was 33.6 ± 5.3 years, and patients were followed up for 129.5 ± 81.9 months. In the NC-APS group, 31 (22.1%) did not fulfill clinical and serological criteria (Subgroup A), 49 (35%) did meet clinical but not serologic criteria (Subgroup B), and 60 (42.9%) fulfilled the serologic criteria but not the clinical ones (Subgroup C). The cardiovascular risk burden was higher in the APS group, due to a higher proportion of smoking. Patients with criteria APS received more intensive treatment than patients in the other study groups. The addition of standard of care (SoC) treatment significantly improved live birth and decreased APO in all groups. Significant clinical differences were observed between the study groups. However, when treated with SoC, fetal–maternal outcomes were similar, with a significant improvement in live births and a decrease in APO. Risk stratification in patients with obstetric morbidity associated with APS can help individualize their treatment.

## 1. Introduction

Antiphospholipid syndrome (APS) is an autoimmune disease characterized by thrombotic and/or obstetric events, associated with the presence of antiphospholipid antibodies (aPLs) [1]. As in all autoimmune diseases, the scientific community, represented by experts in this field, has developed classification criteria in an attempt to standardize research studies at both clinical and research levels [1]. These classification criteria, which are often also used as diagnostic criteria, include the main clinical and serological characteristics of APS and define a very specific patient population. However, in daily clinical practice, physicians face patients who do not strictly meet the classification criteria, but who undoubtedly have the disease [2,3,4,5]. Diagnosing APS requires both clinical and serological criteria, but patients who do not strictly meet the classification criteria may present with what have been called “clinical manifestations related to APS”, or with an inconclusive serological profile not included within the criterion definition. This is especially relevant in the subgroup of patients with obstetric APS [6]. In this regard, in a large multicenter study, Alijotas-Reig et al. [6] have recently described three subgroups of patients with clinical and serological manifestations included in the so-called “non-criteria (NC)-APS”. The stratification of these patients into well-defined subgroups can help to carry out more homogeneous studies in the future and allows a better understanding of the different presentations of the obstetric APS spectrum. In recent years, a growing number of studies (Table 1) have addressed this concept, although design differences have complicated their interpretation [6,7,8,9,10,11,12,13,14,15,16,17,18,19,20]. Interestingly enough, the obstetric outcome of patients with NC-APS receiving the standard of care (SoC) therapy with low-dose aspirin (LDA) and/or low molecular weight heparin (LMWH) is very similar to that of APS patients [6,7,13,18,20]. To further complicate this scenario, Rodríguez-Garcia et al. [15], introduced, in 2012, the concept of “seronegative (SN)-APS”, to refer to those patients who presented clinical manifestations typical of the disease but in whom the serological studies with aPLs included in the classification criteria were persistently negative. However, a growing number of studies have shown that a significant proportion of the so-called SN-APs are carriers of other aPLs (anti-phosphatidylserine/prothrombin, anti-phosphatidyl-ethanolamine, etc.) [21,22,23]. Although today, these patients cannot be classified according to the criteria as APS, it has been suggested that they should be treated according to the same recommendations [24]. All this information has been further complicated by the inclusion, in many of the studies, of patients with other autoimmune diseases, especially systemic lupus erythematosus (SLE) [6,7,12,13,14,15,17,18,19,20].

Taking into account these considerations, our study aimed to analyze the main clinical characteristics of the different subgroups of NC-APS and to compare them with patients with APS and SN-APS, in a cohort of patients from a single center and without other associated systemic autoimmune diseases. Moreover, the comorbidities, serological characteristics, treatments used, and the obstetric outcome are also described.

## 2. Materials and Methods

### 2.1. Study Participants

This retrospective cohort study included 290 consecutive ever-pregnant women followed at the Autoimmune Diseases Pregnancy Clinic, a multidisciplinary unit of a teaching tertiary care hospital between 2005 and 2020. As shown in Table 2, 263 patients were categorized into the following groups: (a) Criteria APS (*n* = 66): patients were classified according to the Sidney classification criteria [1]; (b) NC-APS (*n* = 140): patients who do not meet strict clinical and serological classification criteria for the disease. According to Alijotas-Reig et al. [6], these patients were divided into the following subgroups: Subgroup A (*n* = 31): non-criteria obstetric morbidity related to APS and inconclusive serology; Subgroup B (*n* = 49): clinical manifestations included in the criteria and inconclusive serology; and Subgroup C (*n* = 60): non-criteria obstetric morbidity related to APS and serology included in the classification criteria.; (c) SN-APS (*n* = 57): clinical manifestations included in the criteria and persistently negative serology. Women who fulfilled the classification criteria for rheumatic autoimmune diseases other than APS were excluded.

The information collected from individual cases was completely anonymized, and the study was approved with a waiver of informed consent by the Ethics Committee of Cantabria (internal code: 2021.037) because this is a retrospective clinical record review study. The study conformed to the principles of the Declaration of Helsinki. 

### 2.2. Data Collection

Data were collected using a prespecified standardized questionnaire, in a computerized database. We assessed the following clinical variables:

- Demographic and general characteristics: age, sex, body mass index (BMI), current/past tobacco use, high blood pressure (equal or greater than 140/90 mm Hg or being on antihypertensive agents) [25], dyslipidemia (serum total cholesterol or triglyceride levels greater than 230 mg/dL and 150 mg/dL, respectively or being on lipid-lowering drugs) [26], diabetes mellitus (according to the ADA criteria) [27], past or present family (<50 years) or personal history of thrombotic disease.
- Standard-of-care (SoC) treatment included LDA and/or LMWH. Patients without a previous history of thrombotic events were treated with prophylactic LMWH. Those with a previous thrombotic event did receive therapeutic doses.
- Comorbidities: the three main entities associated with pregnancy outcomes were also recorded; (a) inherited thrombophilia (factor V Leiden, prothrombin mutation, protein S and/or protein C deficiency); (b): thyroid disease (history of hypo/hyperthyroidism or the presence of confirmed specific autoantibodies); (c) obstetric comorbidity (local uterine abnormalities, endometriosis, and polycystic ovary syndrome).

### 2.3. Autoantibody Assessment

The presence of the following antibodies and aPL isotypes was quantified by commercial enzyme immunoassay in solid phase (ELISA; Orgentec Diagnostika GmbH, Mainz, Germany): anticardiolipin antibodies (aCLs) and anti-beta2 glycoprotein I antibodies (AB2GPI) of the IgG and IgM isotypes. The results are reported as quantitative and semiquantitative values. Thus, aCLs are quantified in GPL (aCL IgG; cut off ≤ 6) or MPL (aCL IgM; cut off ≤ 6) according to the standard curve constructed in each test with 5 dilution points of the Harris/Sapporo standards. AB2GPI are quantified as U/mL (cut off ≤ 4). Only medium–high titers of aPLs were considered positive. The criteria recommended by the International Society of Thrombosis and Hemostasis (ISTH) Scientific and Standardization Committee (ISTH) for the standardization of lupus anticoagulant/antiphospholipid antibodies (LA/APA) were applied for the characterization of LA [28,29,30]. Inconclusive serology was defined as persistent low-titer aCL or AB2GPI and/or intermittent AL, aCL, or AB2GPI.

### 2.4. Pregnancy Morbidity Definitions

- Obstetric manifestations: (a) Sidney criteria [1]; (b) Non-criteria obstetric morbidity related to APS: 1–2 early pregnancy losses (<10 weeks), preterm birth (between 34 and 36 + 6 weeks), late preeclampsia (>34 weeks), abruptio placentae and unexplained in vitro fertilization failures (>2) [2];
- Pregnancy loss: early pregnancy loss (<10 weeks) and/or fetal death (>10 weeks);
- Adverse pregnancy outcome (APO): early pregnancy loss, fetal death, preeclampsia, abruptio placentae, and preterm birth (<37 weeks).

### 2.5. Statistical Analysis

Results were expressed as numbers (percentage), mean ± standard deviation (SD) or median and interquartile range (IQR), as appropriate. Student’s t-test or Mann–Whitney U-test or one-way ANOVA were used to compare quantitative variables and Chi-squared or Fisher test, to compare categorical data. A two-tailed *p*-value < 0.05 was considered statistically significant in all the calculations.

## 3. Results

### 3.1. General Features of the Study Cohort

During the study period, 263 consecutive patients fulfilled the inclusion criteria. The main characteristics of the study cohort (Table 3), their serological profile (Table 4), and SoC treatment (Table 5) are shown. The mean age of the overall group was 33.6 ± 5.3 years and the patients were followed up for 129.5 ± 81.9 months (115 (65–195)). The 263 women had 1013 pregnancies, with a mean of 3.9 ± 1.7 (4 (3.0–5.0)) pregnancies per patient.

**Figure 1:**
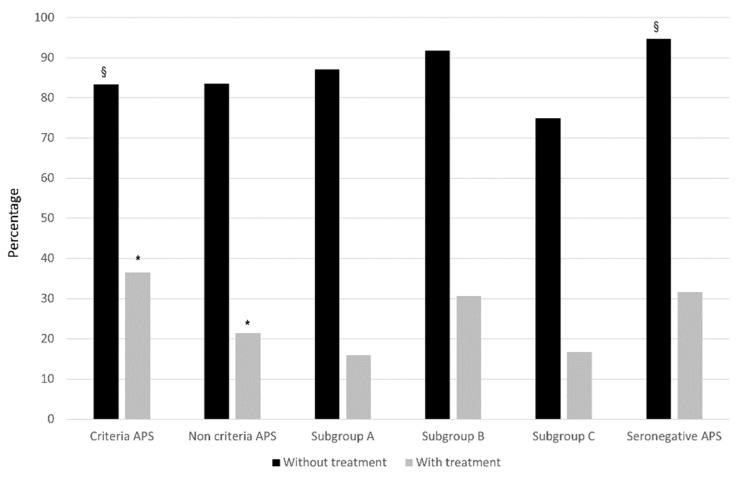
Pregnancy loss in the different groups according to treatment. Rates of patients with pregnancy loss (early pregnancy loss (<10 weeks) and/or fetal death (>10 weeks)) are expressed as percentages in the different groups according to standard (SoC) treatment. * Criteria APS vs. NC-APS: *p* = 0.023; ^§^ Criteria APS vs. SN-APS: *p* < 0.05.

**Figure 2:**
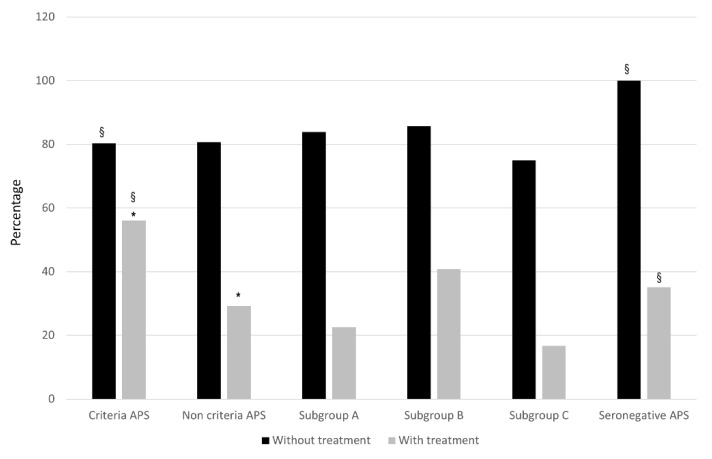
Adverse Pregnancy Outcomes (APO) in the different groups according to treatment. Rates of patients with APO are expressed as percentages in the different groups according to standard (SoC) treatment. Adverse pregnancy outcome (APO) includes early pregnancy loss, fetal death, preeclampsia, abruptio placentae, and preterm birth (< 37 weeks). * Criteria APS vs. NC-APS: *p* < 0.0001; ^§^ Criteria APS vs. SN-APS: *p* < 0.05.

Overall, and despite being a population of young women of childbearing age, the prevalence of cardiovascular risk factors ranged from 44% to 61%, especially in patients with APS. In addition, the most frequent comorbidities with a potential impact on the obstetric outcome, such as hereditary thrombophilia, thyroid disease, or obstetric comorbidities, were also frequent in all study groups. From a serological point of view, the aPL carrier groups had a high prevalence of a high-risk phenotype, including double/triple positivity or the isolated presence of LA. After diagnosis, most of them received SoC treatment with LDA and/or LMWH during pregnancies [31,32,33,34]. As expected, patients with classic APS received more intensive treatment than patients belonging to the other study groups (Table 5). As shown in Figure 1 and Figure 2, the addition of SoC treatment significantly improved the obstetric outcome, both achieving a live birth and decreasing APO.

### 3.2. Are APS Criteria Similar to NC-APS?

One hundred and forty women were included in the NC-APS group. This group was divided into 3 subgroups: 31 (22.1%) did not fulfill clinical and serological criteria (Subgroup A), 49 (35%) did meet clinical criteria but not serologic criteria (Subgroup B), and 60 (42.9%) fulfill the serologic criteria but not the clinical ones. Overall, NC-APS patients were very similar to the APS group except for a higher cardiovascular risk burden in the APS group (*p* = 0.029), especially due to a higher proportion of smoking in APS patients (*p* = 0.002) (Table 3). Although AB2GPI positivity was slightly more frequent in the NC-APS group (*p* = 0.079), a high-risk profile, including double/triple positivity and/or LA positivity was very similar in both groups (Table 4). APS patients had more pregnancies than those in the NC-APS group (4.0 (3.0–5.0) vs. 3.0 (2.0–5.0); *p* = 0.016). As expected, overall clinical manifestations related to the classification criteria were more frequent in the APS group than in the NC-APS. This was the case for fetal death (*p* < 0.0001), ≥ 3 abortions (*p* = 0.002), and thrombosis (*p* = 0.001). On the other hand, patients in the NC-APS group had more frequently ≤ 2 early pregnancy losses (*p* < 0.0001) and IVF failures than the APS patients (*p* = 0.012) (Table 6). As shown in Table 5, more patients in the APS group received SoC treatment, including LDA (*p* = 0.021) and combination therapy (*p* < 0.0001). Patients in the NC-APS group were treated more frequently with corticosteroids plus SoC therapy, although these differences did not reach statistical significance (*p* = 0.057). 

**Figure 3:**
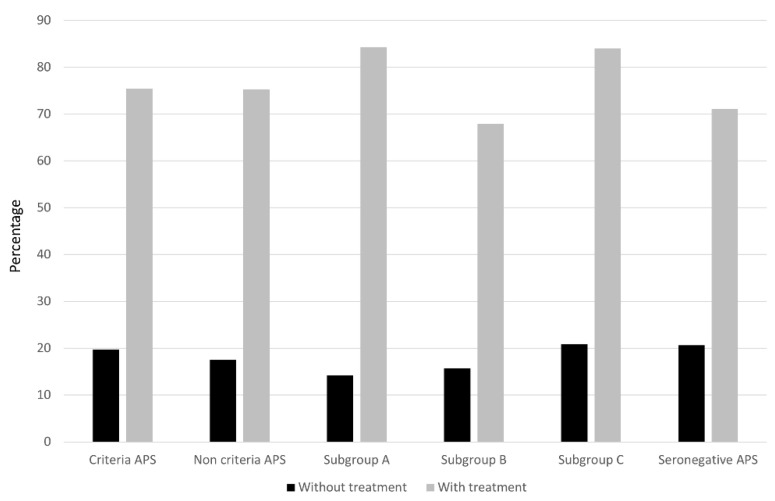
Proportion of successful pregnancy in the study groups after standard treatment. The results show the percentage of live births compared to the number of patients with or without standard (SoC) treatment.

Regardless of the treatment used, the percentage of success with and without treatment in both groups and the live birth rate were very similar between both groups (Table 7 and Figure 3). However, APS patients had a higher rate of fetal losses (*p* = 0.023) and APO (*p* < 0.0001) after receiving SoC therapy (Table 7 and Figure 1 and Figure 2), and this was mainly due to a higher rate of abortion < 10 weeks (*p* = 0.027), fetal death > 10 weeks (*p* = 0.057), and preterm < 37 weeks (*p* = 0.008).

### 3.3. Does It Make Sense to Divide NC-APS into Different Subgroups?

For well-known different reasons, the determination of aPLs is one of the most complex issues of APS [4]. Therefore, we have compared those groups with similar clinical manifestations, but with differences in fulfilling the Sydney serological criteria.

Interestingly enough, subgroup B was almost indistinguishable from the APS group. When compared with subgroup B from a clinical point of view, the difference was only significant for ≥ 3 abortions (*p* = 0.023). The serological profile was also very similar except for a higher frequency of aCLs in the APS group (*p* = 0.01). APS patients received more SoC treatment, including LDA (*p* = 0.01) and combination therapy (*p* = 0.002). The NC-APS group was more frequently treated with corticosteroids plus SoC therapy (*p* = 0.012). To sum up, although we globally found differences between the APS and NC-APS groups, patients in subgroup B were very similar to APS patients, with a similar prognosis but receiving a less complete treatment.

When we compared the subgroups with APS-related obstetric morbidity, subgroups A and C, regardless of whether the Sydney serological criteria were met, both groups of patients were indistinguishable.

### 3.4. Is SN-APS a Different Disease?

Fifty-seven women were included in the SN-APS group. Again, these patients were very similar to the APS group except for a higher cardiovascular risk burden in APS patients (*p* = 0.14), especially due to a higher proportion of smoking in this group (*p* = 0.03) (Table 3). APS patients had fewer pregnancies than those in the SN-APS group (4.0 (3.0–5.0) vs. 5.0 (4.0–6.0), *p* = 0.013). As expected, overall clinical manifestations related to the classification criteria were very similar in both groups, except for a significant increase of ≥3 abortions (*p* = 0.003) in the SN-APS group. Regarding APS-related morbidity, patients in the SN-APS group had fewer late pre-terms than the APS patients (*p* = 0.03) (Table 6). As shown in Table 5, more patients in the APS group received SoC treatment, including LDA (*p* < 0.0001) and combination therapy (*p* < 0.0001). However, patients in the SN-APS group were treated more frequently with corticosteroids plus SoC therapy (*p* < 0.0001). Whatever the therapeutic scheme, the percentage of success with and without treatment in both groups and the live birth rate were again very similar between both groups (Table 7 and Figure 3). However, patients with SN-APS had a higher rate of fetal losses (*p* = 0.047) and APO (*p* < 0.0001) before treatment, but a significant improvement in APO after receiving SoC therapy compared with APS patients (*p* = 0.02). (Table 7, and Figure 1 and Figure 2).

## 4. Discussion

In the present study, we analyze the main clinical characteristics, comorbidities, serological profile, treatment scheme, and obstetric outcome in a large cohort of patients belonging to the obstetric APS spectrum, including NC-APS and SN-APS. Despite the differences inherent in the definition of the different groups, the obstetric prognosis of patients on SoC therapy is very similar and overall satisfactory.

As in obstetric APS, most clinical manifestations occur additively over time and with new pregnancies, one can argue that differences in the follow-up or the number of pregnancies may be relevant when stratifying the study groups. In this regard, although the follow-up was slightly lower in NC-APS patients, especially in subgroups A and C, the differences were not statistically significant. However, the number of pregnancies was significantly lower in subgroups A and C, which do not strictly meet the clinical classification criteria. Thus, it may be possible that if the patients in these two subgroups had a new pregnancy and were not adequately treated, they might easily change to a different study subgroup. This could have been the case in subgroup B and in SN-APS patients, which are the ones that have had a greater number of pregnancies. Thus, it is possible that the patients have been deprived of effective treatment, in the case of subgroup B (since the serological criteria were not strictly met) or in the SN-APS group (due to the complete lack of serological evidence). As shown in Table 7, patients in subgroup B and especially those with SN-APS had a higher frequency of fetal losses and APO before therapy compared to APS patients. Similarly, SoC therapy significantly improved obstetric outcomes in these two groups compared to patients with APS, confirming its efficacy as previously described by other investigators [11,12,17,18]. 

Another relevant aspect concerning APS is whether the serological definition of the criteria is equally adequate for thrombotic and obstetric manifestations. In this sense, some authors have suggested that the persistence of low-aPL titers in patients with obstetric APS has a similar pathogenic value to the presence of medium or high titers [13,35,36,37]. In this study, the presence of low titers has been considered an inconclusive serology, and obviously, a different assessment of aPL titers could also alter the distribution of the different subgroups. Furthermore, several studies have shown that SoC treatment in patients with low-aPL titers led to favorable obstetric outcomes [13,14,16].

The dilemma arises when, given the favorable results of SoC treatment in this and other series of patients [6,11,12,17,19], one wonders whether all patients with suspected APS should receive the same treatment scheme. In our opinion and for several reasons, the answer is probably no. Firstly, there is no clear evidence that combined therapy with ASA and LMWH can be useful in all clinical situations related to APS [37]. Thus, some data can support the use of ASA in monotherapy in unexplained recurrent abortions [38], although this evidence is controversial, even in aPL carriers [39,40,41,42]. Moreover, there are more than reasonable doubts about the use of monotherapy or combined SoC therapy in placentation disorders or implantation failure after IVF techniques [43,44,45,46,47]. While combined therapy with LDA and LMWH has not been proven to be effective in preventing placentation disorders in APS patients [48,49,50], LDA is the only effective treatment, which delays the gestational age at delivery with preeclampsia in the general population [51,52]. Secondly, and despite scant scientific evidence, it is possible that, in a significant proportion of patients who do not strictly meet the APS classification criteria, ASA monotherapy may be more than sufficient to achieve a favorable obstetric outcome ([10] and personal unpublished data). Moreover, another factor to take into account in patients who do not strictly fulfill the classification criteria is the presence of certain manifestations related to the APS [19,53]. Thus, patients with a suggestive clinical picture, but inconclusive serology, who present associated manifestations, such as livedo reticularis or thrombocytopenia, may be candidates for more intensive treatment. However, to date, there is not enough body of evidence to justify this approach. Thirdly, in addition to the clinical and serological profile, factors related to the prognosis of pregnancy itself, such as the age at conception, should be considered [26,54]. Thus, it is more than reasonable to think that, in a given clinical situation, therapeutic options will be determined by maternal age. For example, women with one or two early abortions and conclusive APS serology will receive more intensive treatment if they are older. This is our experience, and the one described by some other experts in the field [4]. Finally, other therapeutic schemes used in patients with recurrent abortions and implantation failure, such as low-dose corticosteroids for a limited period, may be useful in certain obstetric manifestations [55,56]. This is reflected by the more frequent use of low-dose corticosteroids in patients with NC-APS and SN-APS in our cohort, or even in those with refractory APS [57,58,59].

Our study has certain limitations. First of all, those inherent to a retrospective design. Moreover, it is carried out in a single center and a multidisciplinary unit specifically devoted to the treatment of obstetric complications in patients with autoimmune diseases. This means that the results cannot be extrapolated to other populations, and probably to the care of pregnant patients outside specialized units. Finally, other aPLs not included in the classification criteria were not analyzed, which could have helped to better categorize the different groups, especially SN-APS and NC-APS.

We consider that our study has several advantages over previous ones. Firstly, these studies have been carried out in patients with aPLs associated with other autoimmune diseases, mainly SLE [6,7,12,13,14,15,17,18,19,20], whereas those patients have been excluded from our study. Thus, we could analyze a more homogeneous population of patients belonging to the clinical spectrum of APS. Secondly, the present cohort represents the whole spectrum of patients with a clinical suspicion of APS. It ranges from SN-APS to patients with primary APS, defined according to the classification criteria [1]. Moreover, our study includes patients with aPLs who present obstetric manifestations not included in these criteria but represent a very relevant subgroup in routine clinical practice. Another advantage of this study is that, in addition to the cardiovascular risk factors and the serological profile, we have also assessed other comorbidities that could influence the overall obstetric prognosis [60,61,62,63]. 

We believe that the initiative of Alijotas-Reig et al. [6] of dividing patients with the APS clinical/serological spectrum into well-defined subgroups should allow for the development of well-designed clinical trials that include not only patients with APS defined by the classification criteria, but also the remaining patients who represent a very relevant proportion of those managed in real-world clinical practice. It is possible that adequate risk stratification not only for the presence or absence of aPLs but also for key factors such as age, previous obstetric history, or the presence of other comorbidities or clinical manifestations related to APS, could help to better stratify the treatment of future pregnancies. In this sense, the development of a specific score for pregnancy could contribute to a more efficient and homogeneous management of these patients [26].

In summary, as expected, significant clinical differences were observed between the study groups. However, when treated with SoC, fetal–maternal outcomes were similar, with a significant improvement in live births and a decrease in APO. Risk stratification in patients with obstetric morbidity associated with APS can help individualize their treatment.

## Figures and Tables

**Table 1:** Main studies on non-criteria obstetric antiphospholipid syndrome.

Authors [Ref.]	Year	Design	Setting	Group (N)	Other CTD	Main Results	Comments
**Pires da Rosa G et al. [19]**	2022	Retrospective	Multicentric (2)	SN-APS (82) Subgroup B (88)	12–18%	- Similar clinical features and treatment rates - No differences in recurrences	
Spinillo A et al. [20]	2021	Prospective	Single-center	APS (62) NC-APS (48)	31.3%	- High rate of stillbirths and APO in APS - CTD associated with APO	Low-risk subjects are responsible for a high burden of APO
Pregnolato F et al. [13]	2021	Retrospective	Single-center	Criteria APL (100) Low titer APL (55)	36–39%	- Women with low-titer aPLs benefited from SoC therapy	Antiphospholipid antibodies at low titers significantly impact the probability of pregnancy morbidity
Li X et al. [12]	2021	Prospective	Single-center	AP (34) NC-APS (94)	7%	- Similar rates of live births in both groups	Although only 7% had a CTD, 65% of the patients receive CS or IS therapy
Alijotas Reig J et al. [6]	2020	Retrospective Prospective	Multicentric (30)	APS (1000) NC-APS (640)	25–32%	- Obstetric complications were higher in APS - Live births, treatment rates, and thrombotic complications were similar in both groups after SoC	Categorized 3 subgroups [A (27%), B (27%), C (45%)] within the NC-APS
Xi F et al. [18]	2020	Prospective	Single-center	APS (44) NC-APS (91)	Included	- Similar live births rate and good obstetric outcomes with SoC - IUGR more frequent in APS	Although the number of CTD patients was low, 48–60% used corticosteroids and 7–34% used antimalarials
Abisror N et al. [11]	2020	Retrospective	Multicentric (14)	APS (285) SN-APS (187)	Excluded	- Live births and treatment rates were similar in both groups	No differences in patients who received LDA or LDA + LMWH
Lo HW et al. [9]	2020	Retrospective	Single-center	APS (12)	Excluded	- APO more frequent in APS - Live birth rates were lower in NC-APS	Small number of patients
Fredi M et al. [8]	2018	Retrospective	Multicentric (3)	APS (85)	Excluded	- Patients with NC-APS received less LDA + LMWH and also had less APO	In NC-APS, APO was associated with LA and AB2GPI IgG
Ofer-Shiber S et al. [17]	2015	Retrospective	Single-center	APS (126) NC-APS (117)	46%	- Live births and treatment rates were similar in both groups	NC-APS includes persistent aPL titer
Gardiner C et al. [14]	2013	Retrospective	Single-center	APS (100) NC-APS (45)	13.1%	- Low-titer thrombotic APS: 19 - Low-titer obstetric APS: 26	The authors suggest that low-titer aCLs and ab2GPI should be included in the laboratory criteria for the diagnosis of obstetric APS.
Del Ross T et al. [7]	2013	Retrospective	Single-center	aPL carriers (65) NC-APS (74)	47%	- Rate of pregnancy failure in the aPL-positive women not fulfilling obstetrical APS criteria did not improve significantly with LDA therapy	Low-risk population.
Mekinian A et al. [16]	2012	Retrospective	Single-center	APS (25) NC-APS (32)	Excluded	- No differences in thrombotic or obstetric events	Small number of patients
Rodríguez-Garcia JL et al. [15]	2012	Retrospective	Single-center	APS (87) SN-APS (67)	24–44%	- Similar rate of obstetric manifestations - SN-APS had a lower rate of live births	
Sugiura-Ogasawara M et al. [10]	2008	Retrospective	Single-center	NC-APS (68)	Excluded	- High rate of live births with LDA alone	The study includes patients with recurrent pregnancy loss and intermittent aPLs (group B)

CTD: connective tissue disorders; APS: antiphospholipid syndrome; NC-APS: non-criteria APS; aPLs: antiphospholipid antibodies; Group A: obstetric morbidity related to APS and NC-aPL; Group B: obstetric criteria and NC-aPL; Group C: obstetric morbidity related to APS and criteria aPL; CS: corticosteroids; IS: immunosuppressive agents; LDA: low-dose aspirin; LMWH: low molecular weight heparin; APO: adverse pregnancy outcome; SN-APS: seronegative antiphospholipid syndrome; LA: lupus anticoagulant; AB2GPI: anti-beta 2 glycoprotein I; aCLs: anticardiolipin antibodies; SoC: standard of care.

**Table 2:** Study groups according to the clinical and serological manifestations of the Sydney criteria and the presence of obstetric morbidity related to antiphospholipid syndrome (APS) *.

Serology	Clinical Manifestations		
	Sidney Criteria	Related Obstetric Morbidity	No Manifestations
Sidney criteria	Criteria APS (*n* = 66)	Subgroup C (*n* = 60)	Asymptomatic carriers (*n* = 11)
Inconclusive	Subgroup B (*n* = 49)	Subgroup A (*n* = 31)	(*n* = 5)
Negative	Seronegative APS (*n* = 57)	(*n* = 11)	-

* Definitions for related obstetric morbidity and inconclusive serology are defined in Section 2.3 and Section 2.4.

**Table 3:** Demographic characteristics, cardiovascular risk factors, and main comorbidities in the different study groups.

	Criteria APS	Non-Criteria APS	Subgroup A	Subgroup B	Subgroup C	Seronegative APS
Age (years), m ± SD	33.7 ± 4.8	33.6 ± 5.5	33.9 ± 5.0	33.9 ± 5.3	33.3 ± 6.1	33.5 ± 5.5
Time to diagnosis, median (IQR)	30 (13.7–56.3)	20.5 (9.3–39.8)	22 (12–36)	27 (12–67)	12 (6–26.8)	24 (13.5–52.5)
Follow-up (months), m ± SD	121 (80.5–205.5)	108 (51–92)	110 (59–192)	108 (66.5–173)	99.5 (35.3–202)	118 (81.5–170)
FH of thrombosis, %	12.1	**10 ^#^**	3.2	18.4	6.7	**21.1 ^#^**
**CV risk factors, %**	60.6 *	44.3 *	38.7	44.9	45.9	47.4
Obesity	21.3	14.4	23.1	13.3	11.1	15.1
Smoking	47 * ^,**§**^	30.7 *	16.1	34.7	35	**28.1 ^§^**
High blood pressure	9.1	8.6	6.5	8.2	10	7
Diabetes	3	1.4	0	2	1.7	1.8
Dyslipidemia	6.1	5.7	3.2	8.2	5	1.8
**Comorbidities, %**						
Hereditary thrombophilia	18.5	13	17.9	14.6	9.1	21.6
Thyroid disease	12.1	11.4	3.2	14.3	13.3	15.8
Obstetric comorbidity	10.6	17.1	22.6	16.3	15	8.8

APS: antiphospholipid syndrome. FH: familiar history. CV: cardiovascular. m: mean. SD: standard deviation. IQR: interquartile range. * Criteria APS vs. NC-APS: *p* < 0.05; ^§^ Criteria APS vs. SN-APS: *p* < 0.05; ^#^ SN-APS vs. NC-APS: *p* < 0.05.

**Table 4:** Main serological groups.

	Criteria APS	Non-Criteria APS	Subgroup A	Subgroup B	Subgroup C	Seronegative APS
aCL+ (%)	**30.3 ^¶^**	22.1	29.0	**10.2 ^¶^**	28.3	0
AB2GPI+ (%)	19.7	31.4	32.2	26.5	35.0	0
LA+ (%)	18.2	15	9.7	24.5	10.0	0
Double/Triple + (%)	31.8	31.4	29.0	38.8	26.7	0
High-risk aPL profile (%)	50.0	46.4	38.7	63.3	36.7	0

APS: antiphospholipid syndrome. aCLs: anticardiolipin antibodies; AB2GPI: anti-beta 2 glycoprotein I; LA: lupus anticoagulant; High-risk. aPL profile: Double/Triple+ and/or LA+; aPLs: antiphospholipid antibodies. ^¶^ Criteria APS vs. Group B; *p* = 0.01.

**Table 5:** Main treatments in the different study groups.

	Criteria APS	Non-Criteria APS	Subgroup A	Subgroup B	Subgroup C	Seronegative APS
**Standard treatment, %**						
- LDA	98.5 * ^,**§**,**¶**^	89.3 *	83.9	**85.7 ^¶^**	95	**78.9 ^§^**
- LMWH	75.8 * ^,**§**,**¶**^	49.3 *	45.2	**51 ^¶^**	50	**49.1 ^§^**
- LDA + LMWH	75.8 * ^,**§**,**¶**^	45.7 *	38.7	**46.9 ^¶^**	48.3	**42.1 ^§^**
**Additional treatments, %**						
- Corticosteroids	**1.8 ^§^** ^,**¶**^	**10.8 ^#^**	4.5	**17.1 ^¶^**	8.9	**31.4 ^§,#^**
- Antimalarials	5.4	6.8	4.3	5.7	8.9	0

APS: antiphospholipid syndrome; LDA: low-dose aspirin; LMWH: low molecular weight heparin. * Criteria APS vs. NC-APS: *p* < 0.05; ^§^ Criteria APS vs. SN-APS: *p* < 0.05; ^#^ SN-APS vs. NC-APS: *p* < 0.05; ^¶^ Criteria APS vs. Group B *p* < 0.05.

**Table 6:** Clinical APS subgroups according to the Sydney criteria, obstetric morbidity related to APS, and thrombocytopenia in the different study groups.

	Criteria APS	Non-Criteria APS	Subgroup A	Subgroup B	Subgroup C	Seronegative APS
N of pregnancies, m ± SD median (IQR)	4.0 ± 1.5 4.0 (3.0–5.0) *^,§^	3.4 ± 1.6 3.0 (2.0–5.0) *^,#^	2.7 ± 1.1 3.0 (2.0–4.0)	4.4 ± 1.7 5.0 (3.0–5.0)	2.9 ± 1.3 3.0 (2.0–4.0)	4.8 ± 1.5 5.0 (4.0–6.0) ^§,#^
**Sidney Criteria, %**						
Fetal death > 10 weeks	37.9 *	8.6 *^,#^	0	24.5	0	**22.8 ^#^**
Preterm < 34 weeks	10.6	3.6	0	10.2	0	5.3
Abortion < 10 weeks (≥3)	43.9 *^,§,¶^	22.8 *^,#^	0	**65.3 ^¶^**	0	**70.2 ^§,#^**
Thrombosis	16.7 *	2.8 *	0	8.2	0	7
**Obstetric morbidity, %**						
Abortion < 10 weeks (≤2)	24.2 *	53.6 *^,#^	71	14.3	76.7	**15.8 ^#^**
Preterm 34–37 weeks	**15.2 ^§^**	7.1	12.9	4.1	4.9	**3.5 ^§^**
Preeclampsia/Eclampsia > 34 weeks	6.1	7.9	6.5	4.1	11.7	1.8
Abruptio Placentae	0	3.6	9.7	2.0	1.7	0
IVF failures (>2)	1.5 *	12.1 *	16.1	4.1	16.7	3.5
HELLP	0	3.6	3.2	6.1	1.7	0
**Thrombocytopenia, %**	1.5	2.9	0	2	5	0

APS: antiphospholipid syndrome; N: number; m: mean; IQR: interquartile range; IVF: in vitro fertilization; HELLP: Hemolysis, elevated liver enzymes, and low platelets. * Criteria APS vs. NC-APS: *p* < 0.05; ^§^ Criteria APS vs. SN-APS: *p* < 0.05; ^#^ SN-APS vs. NC-APS: *p* < 0.05; ^¶^ Criteria APS vs. Group B *p* = 0.023.

**Table 7:** Obstetric outcome and main obstetric complications in the different groups according to treatment.

	Criteria APS	Non-Criteria APS	Subgroup A	Subgroup B	Subgroup C	Seronegative APS
**Live births, %** Without treatment With treatment	33.3 75.4	35.0 70.7	25.8 77.4	40.8 61.2	34.4 73.8	47.4 71.9
**Pregnancy loss, %** Without treatment With treatment	**83.3 ^§^** 36.4 *	**83.6 ^#^** 21.4 *	87.1 16	91.8 30.6	75.0 16.7	**94.7 ^§,#^** 31.6
**Adverse pregnancy outcome, %** Without treatment With treatment	**80.3 ^§^** 56.1 *^,§^	**80.7 ^#^** 29.3 *	83.9 22.6	85.7 40.8	75 23.3	**100 ^§,#^** **35.1 ^§^**
**Abortion < 10 weeks, %** Without treatment With treatment	**62.1 ^§^** 33.3 *	**72.9 ^#^** 19.3 *^,#^	77.4 12.9	75.5 28.6	68.3 15.0	**89.5 ^§,#^** **33.3 ^#^**
**Fetal death > 10 weeks, %** Without treatment With treatment	34.8 * 10.6 ^§^	7.1 *^,#^ 3.6	0 0	20.4 10.2	0 0	**19.3 ^#^** **0 ^§^**
**Preeclampsia/Eclampsia, %** Without treatment With treatment	6.1 3.0	5.0 5.0	0 3.2	4.1 4.1	8.3 7	7 1.8
**Preterm < 37 weeks, %** Without treatment With treatment	9.1 19.7 *^,§^	7.1 7.1 *	9.7 9.7	6.1 8.2	7.0 5.0	10.5 **0 ^§^**
**Abruptio placentae, %** Without treatment With treatment	0 0	2.1 2.9	3.2 3.2	0 4.1	3.3 1.7	1.8 0

* Criteria APS vs. NC-APS: *p* < 0.05; ^§^ Criteria APS vs. SN-APS: *p* < 0.05; ^#^ SN-APS vs. NC-APS: *p* < 0.05.

## Data Availability

Due to research still being conducted on the project in our research group full data are not available. Additional data are available upon reasonable request to the corresponding author.
